# Natural Degradation: Polymer Degradation under Different Conditions

**DOI:** 10.3390/polym14173595

**Published:** 2022-08-31

**Authors:** Alexandre A. Vetcher, Alexey L. Iordanskii

**Affiliations:** 1 Institute of Biochemical Technology and Nanotechnology (IBTN) of the Peoples’ Friendship University of Russia (RUDN), 6 Miklukho-Maklaya St., 117198 Moscow, Russia; 2 Complementary and Integrative Health Clinic of Dr. Shishonin, 5 Yasnogorskaya St., 117588 Moscow, Russia; 3 N.N. Semenov Federal Research Center for Chemical Physics, Russian Academy of Sciences, 4 Kosygin St. 4, 119334 Moscow, Russia

Natural degradation (ND) is currently one of the main directions of polymer research. This direction is important for the technological applications of both natural and synthetic polymers and their composites. In some cases, we are interested in increasing their stability, e.g., if we are talking about the decay of intervertebral cartilage, teeth, or the polymer coatings of vehicles and buildings. In others, we are interested in accelerating their decay. The most prominent objects of such applications are polymer carriers for targeted drug delivery, used packaging materials, or non-recoverable car tires. There is a third direction of managed degradation, also. It could be employed in controlled release applications. In all these cases, even ND could occur inside cells or inside a living organism or in the environment under the influence of the degradation-causing factors inherent in it (e.g., ozone, oxygen, and ultraviolet, etc.). It is also obvious that polymers and composites are involved in dissipative synthesis decay type structures that are at least inside a living organism. The contributions to this Special Issue, although not covering the full range of the ND field, give a good overview of some important topics that are required for a sustainable future.

The possibility to apply the theory of centralized aerobic-anaerobic energy balance compensation to the balance of the cervical spine cartilage’s polymeric part in the system of biodegradation and regeneration is the focus of [1]. This contribution employs the thermodynamics of irreversible processes to the above-mentioned system.

Another important topic is the influence of composite components and their structure on ND dynamics. In this Special Issue, we have reports devoted to the influence of Ti_x_Si_y_ oxides on ND of PLA [2], the peculiarities of polyolefins’ ND [3], the effect of small additions (1–5 wt.%) of tetraphenylporphyrin and its complexes with Fe (III) and Sn (IV) on the structure, and subsequently, the ND-associated properties of ultrathin fibers that are based on poly(3-hydroxybutyrate) [4], the combined ultragreen chemical and biocatalytic depolymerization of polyethylene terephthalate (PET) using a deep eutectic solvent-based low-energy microwave treatment followed by an enzymatic hydrolysis to facilitate PET biodepolymerisation [5], the biodegradation and thermo-destruction of films from blends of poly(3-hydroxybutyrate) and chitosan by pouring them from a solution, and nonwoven fibrous materials that are obtained by electrospinning [6], and in the rat-transplanted biodegradation of a single and double cross-linked GelMA hydrogel [7].

Some research studies are devoted directly to waste utilization/recycling, e.g., the application of a mechanochemical method to analyze the recycling mechanism of polyurethane foam and optimize the recycling process, and the regeneration the polyurethane foam powder breaks the C–O bond of the polyurethane foam (in order to greatly enhance the activity of the powder) [8], and also to the wastewater and utilizing its energy potential via the anaerobic digestion of municipality wastewater [9].

The contemporary research is hard to imagine without the creation of a working mathematical model. We have two papers; one is devoted to the development of novel models of the pyrolysis of waste polyvinyl chloride [10]; another is devoted to the modelling of the hygrothermal ageing of epoxy resins and epoxy matrix composite materials [11].

The Special Issue ends with the review on biopolymeric nanoparticles and highlights their various synthesis methods as well as the modulation of their abilities to degrade at different conditions [12]. 

## Data Availability

Not applicable.
